# Typhoid Fever in Breast-Fed Infants

**Published:** 1916-03

**Authors:** 


**Typhoid Fever in Breast-Fed Infants.** Policlinica, Valencia, October 1915. Dr. J. Aguilar Jordán reports two cases. The first a child of 14 months afflicted with noma, pale and ill-nourished. The tongue presented no characteristic features. Abdomen flaccid, spleen enormously enlarged, liver enlarged.

Diarrhoea present with 4-6 green stools daily, and an abundance of mucus. Slight bronchitis, pulse 130, fever intermittent. A diagnosis of malaria was made and quinine given hypodermatically with no results. A splenic puncture was then made, thinking the case was one of Kala-Azar, and the typhoid bacillus was isolated. The second case, 9 months old, had a history of diarrhoea for 15 days. Prostration profound, stupor present and slight symptoms of meningitis. Abdomen moderately tympanitic but no enlargement of liver or spleen. The illness set in with high fever, chill, sweating and the rigor subsided. This repeated itself for several days, then the fever changed from intermittent to remittent. No plasmodia were found but the blood responded strongly to the Widal test. Intestinal hemorrhages are rare in these cases. He calls attention to the fact that breast-fed children are often fed cow's milk, etc., and thus the infection may occur. He does not believe that mother's milk carries the infection, but realizes that typhoid carries may be responsible for some cases. For obvious reasons he considers it prudent to remove the child from the mother or wet nurse should the latter be proved to be carriers of the bacilli.

